# Sarcopenia index based on serum creatinine and cystatin C is associated with 3-year mortality in hospitalized older patients

**DOI:** 10.1038/s41598-020-58304-z

**Published:** 2020-01-27

**Authors:** Tianjiao Tang, Yan Zhuo, Lingling Xie, Haozhong Wang, Ming Yang

**Affiliations:** 1 The Center of Gerontology and Geriatrics, West China Hospital, Sichuan University, No. 37 Guoxue Lane, Chengdu, Sichuan China; 2 Department of Healthcare, West China Hospital, Sichuan University, No. 37 Guoxue Lane, Chengdu, Sichuan China; 3 Department of Orthopedic Surgery, West China Hospital, Sichuan University, No. 37 Guoxue Lane, Chengdu, Sichuan China; 4 Precision Medicine Research Center, West China Hospital, Sichuan University, No. 37 Guoxue Lane, Chengdu, Sichuan China

**Keywords:** Geriatrics, Prognosis, Outcomes research

## Abstract

To investigate the association of the sarcopenia index (SI, serum creatinine value/cystatin C value × 100) with 3-year mortality and readmission among older inpatients, we reanalyzed a prospective study in the geriatric ward of a teaching hospital in western China. Older inpatients aged ≥ 60 years with normal kidney function were included. Survival status and readmission information were assessed annually during the 3-year follow-up. We applied Cox regression models to calculate the hazard ratio (HR) and 95% confidence intervals (CIs) of sarcopenia for predicting mortality and readmission. We included 248 participants (mean age: 81.2 ± 6.6 years). During the follow-up, 57 participants (23.9%) died, whereas 179 participants (75.2%) were readmitted at least one time. The SI was positively correlated with body mass index (BMI) (r = 0.214, p = 0.001), calf circumference (CC) (r = 0.253, p < 0.001), handgrip strength (r = 0.244, p < 0.001), and gait speed (r = 0.221, p < 0.001). A higher SI was independently associated with a lower risk of 3-year all-cause mortality after adjusting for potential confounders (HR per 1-SD = 0.80, 95% CI: 0.63–0.97). The SI was not significantly associated with readmission (HR per 1-SD = 0.97, 95% CI: 0.77–1.25). In conclusion, the SI is associated with 3-year all-cause mortality but not readmission in a study population of hospitalized older patients.

## Introduction

Sarcopenia was traditionally considered a geriatric syndrome characterized by low muscle mass and inadequate mass strength and/or physical performance^1^. It is prevalent in the population older than 65 years, especially in those who are hospitalized or institutionalized^2^, and is associated with an increased risk of adverse outcomes, such as functional decline, poor quality of life and death^3–5^.

Most recently, sarcopenia has been recognized as a disease with an international classification of disease, tenth revision, clinical modification (ICD-10-MC) Code^6,7^. This marks a substantial step forward in the understanding of sarcopenia and evokes the urgent need for a simple but accurate diagnostic method for the detection of sarcopenia.

Most of the current international consensuses agree that the diagnosis of sarcopenia should include low muscle mass and decreased muscle function^6,8–11^. Low muscle mass is usually measured with magnetic resonance imaging (MRI), computed tomography (CT), dual-energy X-ray absorptiometry (DXA) or bioelectrical impedance analysis (BIA). However, these methods are time-consuming, inconvenient, and device-reliable, and some of these methods are expensive and may induce radiation exposure. Decreased muscle function is usually estimated by handgrip strength and gait speed, which also need specific devices or experienced staff. To simplify the detection of sarcopenia, some efforts have been made, such as the SARC-F (a self-report questionnaire including five domains: strength, ambulation, rising from a chair, stair climbing, and history of falls)^12^ and the Ishii formula (a screening formula including three objective variables: age, handgrip strength and calf circumference [CC])^13^.

Several serum biomarkers have been applied as the indicators for sarcopenia as well. For example, serum creatinine is derived from creatine phosphate, its production is affected by muscle mass. Low serum creatinine value has been used as a surrogate of low muscle mass and is associated with adverse outcomes^14,15^. Conversely, cystatin C is a small protein originating from all nucleated cells, its production is relatively constant and less affected by skeletal muscle mass. Therefore, Kashani *et al*. recently used the two biomarkers to develop a novel sarcopenia index (SI, serum creatinine [mg/dl]/cystatin C [mg/l] × 100) for estimating skeletal muscle mass^16^. They reported that the SI was a fair measure for muscle mass and could modestly predict in-hospital mortality among intensive care unit (ICU) patients with normal kidney function. In another study from the same team, the authors reported that the SI was a potentially objective measure for estimating muscle mass among 28 lung transplant patients^17^.

Unlike other complex tools, the SI is only based on the serum concentration of creatinine and cystatin C, which is objective and routinely tested in older people. If the SI can accurately estimate sarcopenia in older people, it would be of great value for simplifying the diagnostic procedure of sarcopenia. Unfortunately, our team compared the SI with four classic diagnostic criteria of sarcopenia and found it could not accurately detect either low muscle mass or sarcopenia in community-dwelling older people^18^. However, most recently, Barreto *et al*.^19^ reported that the SI was a predictor of muscle mass and related to longer hospital length of stay and frailty among 171 ICU patients. This finding got us wondering: is the SI associated with the prognosis of non-ICU older inpatients? Therefore, we reanalyzed the data of a prospective study to evaluate whether the SI is associated with long-term mortality and readmission among hospitalized older adults.

## Methods

We reanalyzed the data of a prospective observational study that was conducted in the acute ward of the Center of Gerontology and Geriatrics, West China Hospital of Sichuan University^20^. The study protocol was approved by the Research Ethics Committee of Sichuan University. All participants were fully informed and written informed consent was obtained from every participant or their legal proxies (for those who could not write their names). All the methods in this study were in accordance with the relevant guidelines and regulations.

### Participants in baseline investigation

The detailed information of the study has been published previously^20^. In brief, between February and August 2012, we consecutively recruited patients aged 60 years and older and agreed to participate in the study. We excluded patients with cachexia or severe diseases that made them fail to communicate with the interviewers or to perform the walking test. Furthermore, for the current analysis, participants should have a record of serum creatinine and cystatin C measured upon admission. We excluded patients with an eGFR of <90 ml/min/1.73 m^2^ at admission from the current analysis as well.

### Data collection

Well-trained interviewers obtained baseline data from all participants through face-to-face interviews within 48 hours of admission. Well-trained technicians performed the anthropometric measurements. All interviewers and technicians received specific training and passed a training test prior to the formal investigation.

### Measurement of cystatin C and creatinine, and the SI

Blood was drawn by experienced nurses in the morning after a fast of more than eight hours. Serum cystatin C concentration (mg/l) was measured with the cystatin C immunoturbidimetric assay (Sichuan Mike Biotechnology Co., Ltd., Chengdu, China), and serum creatinine concentration (mg/dl) was measured with the Roche enzymatic method (Creatinine Jaffe Gen.2 assay: Roche Diagnostics GmbH, Mannheim, Germany). The SI was calculated according to the following equation: serum creatinine value/cystatin C value × 100. According to the SI, all participants were separated into four groups, from Q1 corresponding to the first (lowest) quartile to Q4 corresponding to the fourth (highest) quantile. The quartile cut-off points were as follows: Q1 < 63.9, Q2 63.9–74.3, Q3 74.3–84.2, Q4 > 84.3.

### Measurement of calf circumference (CC), gait speed, handgrip strength

Trained technicians measured the CC by using a millimeter-graded tape around the largest part of the calf to the nearest 0.1 cm, with the participants placed in the supine position, with the left knee raised and the calf placed at a right angle to the thigh. A 4-meter walking test was performed to measure the gait speed^21^. The participants were asked to walk a 4-meter distance with their usual speed on a flat level with no obstructions, and the consuming time was recorded by trained technicians to the nearest of 0.1 s. Gait speed was then calculated by the equation: gait speed (m/s) = 4/consuming time. The participants could use canes or walkers during the walking test, if necessary. The handgrip strength was measured by trained technicians using a handheld dynamometer based on train gauge sensors (EH101, Xiangshan Inc., Guangdong, China) to the nearest 0.1 kg. In a sitting position with the shoulder adducted, the elbow flexed at a 110° angle, the wrist placed in a neutral position, and the interphalangeal joint of the index finger positioned at a 90° angle, the participants were asked to grasp the dynamometer at their maximal power. Both hands were measured three times and the highest value of either hand was recorded for the analyses^9^.

### Assessment of potential covariates

The following covariates were collected from the hospital information systems and the face-to-face interviews: age, sex, smoking status, alcohol drinking status, and comorbidities (hypertension, ischemic heart disease, respiratory disease, acute infection, liver disease, central nervous system [CNS] disease, diabetes, osteoarthritis, tumor of any type, falls in the previous 12 months). The participants’ nutritional status and cognitive function were also evaluated by using the revised version of Mini Nutritional Assessment short-form (MNA-SF)^22^ and the Chinese version of the Mini-Mental Status Examination (MMSE)^23^, respectively. The highest score for the MNA-SF is 14; a score between 8 and 11 indicates that the participant is ‘at risk of malnutrition’; and a score of ≤7 indicates that the participant has malnutrition^22^. Cognitive impairment is defined as an MMSE score of ≤17 for illiterates, ≤20 for primary school graduates, and ≤24 for high school graduates or individuals with higher education^23^. The Chinese version of the 30-item Geriatric Depression Scale (GDS-30) was applied to evaluate depression^24^. A GDS-30 score of ≥11 suggests depression^24^. In addition, hemoglobin and prealbumin were also tested for all participants.

### Follow-up

During the 3-year follow-up, we obtained the survival status and readmission information of the participants by annual telephone interviews. The death events were further confirmed by the data retrieved from the Local Death Registry (but the readmission information was not). The period from the first investigation to the date of death was recorded for the participants who died during the follow-up. The period from the first investigation to the end of the last follow-up was recorded for the participants who did not die. The first readmission time was recorded for the individuals who were readmitted to hospitals several times during the 3-year period of follow-up.

### Statistical analysis

First, the normality of continuous data was investigated using the Kolmogorov–Smirnov test. Then, the data were presented as number (percentage) for categorical variables and mean (standard deviation [SD]) for continuous variables.

For continuous variables, a one-way ANOVA was used to detect differences across groups, and Fisher’s Least Significant Difference (LSD) post hoc analysis was used to determine the difference between every two groups. For categorical variables, the chi-squared test was used to detect the difference across groups. When a significant difference was identified across groups, column proportions tests (z-tests) with Bonferroni correction were performed to determine the difference between every two groups.

Univariate and multivariate Cox proportional-hazard models were applied to calculate the unadjusted and adjusted hazard ratio (HR) and 95% confidence interval (CI) of the SI for mortality and readmission, respectively. The SI was treated as either a continuous variable (per 1-SD) or a categorical variable (using the quartile cut-off points as mentioned above). Age, sex, and other confounders which were significantly different between the quartile groups of SI were adjusted for in the multivariate Cox models. In addition, survival and readmission curves were estimated using the Kaplan-Meier method to determine the impact of quartile of SI on mortality and readmission, respectively. The differences between the curves were compared using log-rank tests.

All data analyses were performed using SPSS version 20.0 (SPSS Inc., Chicago, IL, USA). During most testing, p < 0.05 was considered statistically significant, however, p-values were corrected for the z-tests with the Bonferroni correction (with the statistical significance set at p < 0.008, where 0.008 = 0.05/6).

## Results

### Characteristics of the study population

**Figure 1:**
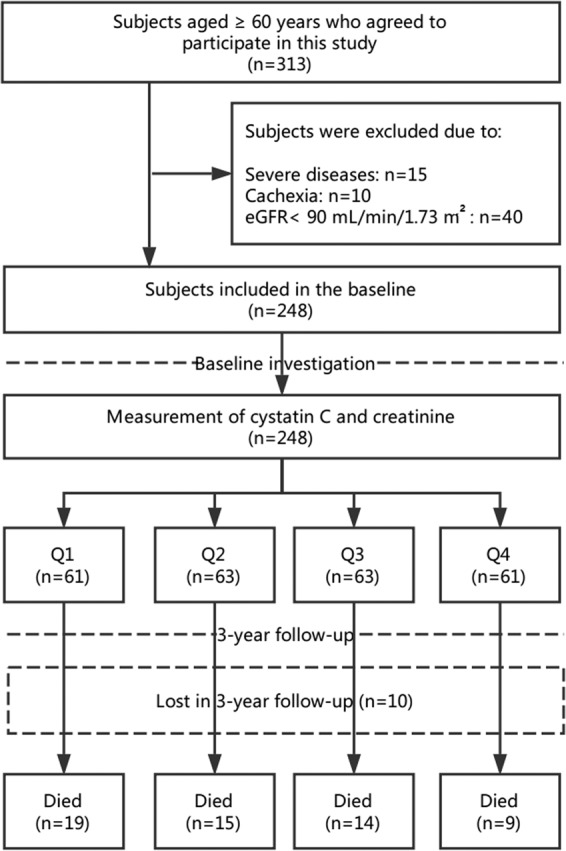
Study flow diagram.

A total of 313 participants agreed to participate in this study, 65 of them were excluded due to different reasons (Fig. 1). As a result, 248 participants (201 men and 47 women; mean age: 81.2 ± 6.6 years) were included in the current baseline analyses. Ten of the 248 participants lost to follow-up during the 3-year follow-up, which led to a final sample size of 238 participants (Fig. 1).

The baseline characteristics of participants according to the quartiles of SI are shown in Table 1. The mean age of the participants in the Q4 group was significantly lower than the other three groups. The prevalence of depression, cognitive impairment, and malnutrition was significantly higher in the Q1 group than in the other three groups, whereas body mass index (BMI), CC in men, gait speed in women, handgrip strength in men, and hemoglobin were the lowest in the Q1 group.

**Table 1:** Baseline characteristics of participants according to the quartiles of sarcopenia index.

Characteristics	Total (n = 248)	Quartiles of sarcopenia index				
		Q1 (n = 61)	Q2 (n = 63)	Q3 (n = 63)	Q4 (n = 61)	p-value
Age (years)	81.2 (6.6)	81.3 (5.7)^d^	83.0 (6.2)^d^	81.9 (5.8)^d^	78.3 (7.6)^a–c^	<0.001
Men (%)	201 (81.0)	41 (67.2)	50 (79.4)	54 (85.7)	56 (91.8)	0.004
Current smokers (%)	26 (10.5)	5 (8.2)	8 (12.7)	7 (11.1)	6 (9.8)	0.868
Current alcohol drinkers (%)	32 (12.9)	5 (8.2)	7 (11.1)	9 (14.3)	11 (18.0)	0.404
**Comorbidities (%)**						
Hypertension	152 (61.3)	39 (63.9)	45 (71.4)	37 (58.7)	31 (50.8)	0.116
Ischemic heart disease	87 (35.1)	22 (36.1)	28 (44.4)	20 (31.7)	17 (27.9)	0.245
Respiratory disease	69 (27.8)	19 (29.5)	19 (30.2)	16 (25.4)	16 (26.2)	0.915
Acute infection	57 (23.0)	12 (19.7)	15 (23.8)	14 (22.2)	16 (26.2)	0.853
Liver disease	22 (8.9)	3 (4.9)	7 (11.1)	5 (7.9)	7 (11.5)	0.542
CNS disease	15 (6.0)	4 (6.6)	5 (7.9)	3 (4.8)	3 (4.9)	0.863
Diabetes	70 (28.2)	16 (26.2)	21 (33.3)	18 (28.6)	15 (24.6)	0.721
Osteoarthritis	68 (27.4)	17 (27.9)	18 (28.6)	17 (27.0)	16 (26.2)	0.992
Tumor of any type	38 (15.3)	14 (23.0)	7 (11.0)	9 (14.3)	8 (13.1)	0.275
Falls in the previous 12 months	36 (14.5)	4 (6.6)	12 (19.0)	9 (14.3)	11 (18.0)	0.190
Depression	58 (23.4)	23 (37.7)^b–d^	16 (25.4)^a^	7 (11.1)^a^	12 (19.7)^a^	0.005
Cognitive impairment	86 (34.7)	32 (52.5)^b–d^	22 (34.9)^a^	19 (30.2)^a^	13 (21.3)^a^	0.003
**Nutrition status (%)**						
At risk of malnutrition	104 (41.9)	27 (44.3)	28 (44.4)	26 (41.3)	23 (37.7)	0.006
Malnutrition	27 (10.9)	14 (23.0)^b–d^	7 (11.1)^a^	4 (6.3)^a^	2 (3.3)^a^	
BMI (men, kg/m^2^)	22.7 (3.6)	21.5 (3.2)^c,d^	22.5 (3.7)	23.1 (3.9)^a^	23.4 (3.1)^a^	0.053
BMI (women, kg/m^2^)	21.9 (3.8)	20.7 (2.4)^d^	21.4 (3.0)^d^	22.6 (5.2)	26.5 (5.2)^a,c^	0.013
CC (men, cm)	32.7 (3.7)	31.6 (3.7)^d^	32.1 (3.8)^d^	32.9 (3.9)	33.8 (3.2)^a,b^	0.032
CC (women, cm)	31.9 (4.7)	30.2 (4.1)	30.4 (5.2)	31.2 (5.3)	33.6 (1.8)	0.090
Gait speed (men, m/s)	0.78 (0.43)	0.66 (0.24)	0.78 (0.31)	0.80 (0.30)	0.85 (0.49)	0.091
Gait speed (women, m/s)	0.70 (0.30)	0.55 (0.18)^c,d^	0.70 (0.24)	0.89 (0.37) ^a^	0.96 (0.20)^a^	0.001
Handgrip strength (men, kg)	22.2 (8.6)	19.0 (7.7)^d^	20.3 (7.9)	23.0 (10.1)	25.8 (6.9)^a^	<0.001
Handgrip strength (women, kg)	14.7 (7.3)	12.9 (7.0)^d^	13.2 (7.4)	16.2 (4.4)	19.9 (7.6)^a^	0.089
Hemoglobin (g/l)	123.9 (23.6)	113.5 (21.9)^b–d^	126.5 (27.8)^a^	128.2 (17.2)^a^	127.4 (23.8)^a^	0.001
Prealbumin (mg/l)	201.0 (53.8)	195.6 (69.3)	197.1 (52.5)	210.0 (34.9)	202.1 (53.8)	0.821

Data are presented as the number (percent) for the following variables: women, current smokers, current alcohol drinkers, specific comorbidities, and nutrition status. For the other variables, the mean (SD) is used.

For continuous variables, one-way ANOVA was used to detect differences across groups for the continuous variables, and Fisher’s Least Significant Difference (LSD) post hoc analysis was used to determine the difference between every two groups. For the categorical variables, the chi-squared test was used to detect the difference across groups. When significant difference was identified across groups, column proportions tests (z-tests) with Bonferroni correction were performed to determine the difference between every two groups.

During most testing, p < 0.05 was considered statistically significant, however, p-values were corrected for z-tests with the Bonferroni correction (with the statistical significance set at p < 0.008, where 0.008 = 0.05/6).

Q stands for sarcopenia index: Q1 is the lowest quartile and Q4 is the highest quartile. Cutoffs for sarcopenia index are Q1 < 63.9, Q2 63.9–74.3, Q3 74.3–84.2, Q4 > 84.3.

BMI: body mass index; CC: calf circumference; CNS: central nervous system; SMI: skeletal muscle index.

^a^Significantly different from the Q1 group.

^b^Significantly different from the Q2 group.

^c^Significantly different from the Q3 group.

^d^Significantly different from the Q4 group.

### Correlation between the SI and BMI, CC, handgrip strength, and gait speed

**Figure 2:**
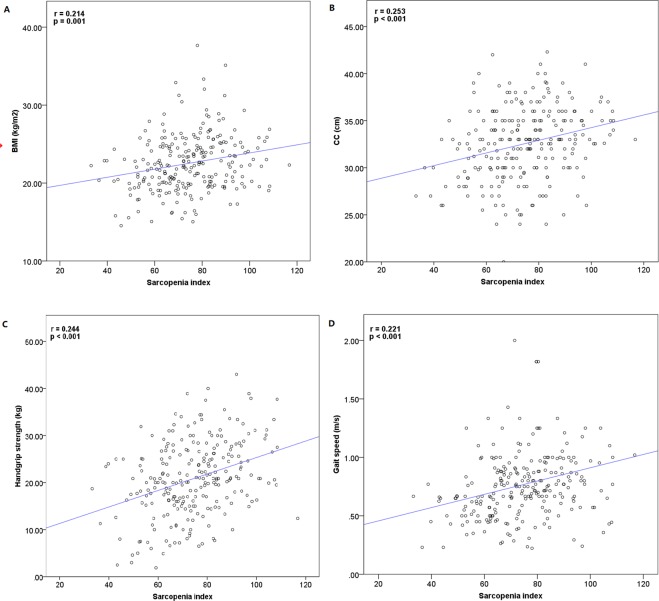
Association between sarcopenia index and BMI (**A**), CC (**B**), handgrip strength (**C**), and gait speed (**D**) at the baseline investigation.

The SI was positively correlated with BMI (r = 0.214, p = 0.001), CC (r = 0.253, p < 0.001), handgrip strength (r = 0.244, p < 0.001), and gait speed (r = 0.221, p < 0.001) (Fig. 2).

### Association between the SI and 3-year all-cause mortality

During the 3-year follow-up, a total of 57 participants (23.9%) died. The mortality according to the quartiles of the SI was 32.2%, 25.0%, 23.3% and 15.3% in the Q1, Q2, Q3, and Q4 groups, respectively.

When the SI was treated as a continuous variable (per 1-SD), a higher SI was independently associated with a lower risk of 3-year all-cause mortality after adjusting for potential confounders (HR per 1-SD = 0.80, 95% CI: 0.63–0.97) (Table 2).

**Table 2:** Association between sarcopenia and mortality (3-year follow-up) according to Cox Regression Models adjusted for potential confounders.

	Unadjusted	Model 1	Model 2	Model 3
Sarcopenia index per 1-SD	0.77 (0.59–0.98)	0.78 (0.59–0.97)	0.81 (0.65–0.95)	0.80 (0.63–0.97)
Quartile of sarcopenia index				
Q1	2.31 (1.25–5.11)	2.10 (1.18–4.71)	1.98 (1.12–4.35)	1.85 (1.10–4.21)
Q2	1.71 (1.01–3.89)	1.46 (1.05–3.16)	1.39 (1.08–3.01)	1.28 (1.03–3.11)
Q3	1.54 (0.67–3.58)	1.50 (0.74–3.10)	1.45 (0.88–2.89)	1.25 (0.78–2.56)
Q4	1 (reference)	1 (reference)	1 (reference)	1 (reference)

Data are presented as hazard ratios (95% confidential intervals). Sarcopenia index was treated as both a categorical variable (using quartile cutoff points) and a continuous variable (per 1-SD), separately.

Q stands for sarcopenia index: Q1 is the lowest quartile and Q4 is the highest quartile. Cutoffs for sarcopenia index are Q1 < 63.9, Q2 63.9-74.3, Q3 74.3-84.2, Q4 > 84.3.

Model 1: adjusted for age and gender. Model 2: adjusted for age, gender, depression and cognitive impairment. Model 3: adjusted for age, gender, depression, cognitive impairment, nutrition status, hemoglobin, body mass index, calf circumference, gait speed, and handgrip strength.

We also evaluated the association between different quartiles of SI and 3-year all-cause mortality. After adjusting for potential confounders, the Q1 group and the Q2 group were associated with 3-year all-cause mortality (HR = 1.85, 95% CI: 1.10–4.21; and HR = 1.28, 95% CI: 1.03–3.11, respectively) when using the Q4 group as the reference. The Q3 group was not significantly associated with 3-year all-cause mortality (HR = 1.25, 95% CI: 0.78–2.56) (Table 2).

**Figure 3:**
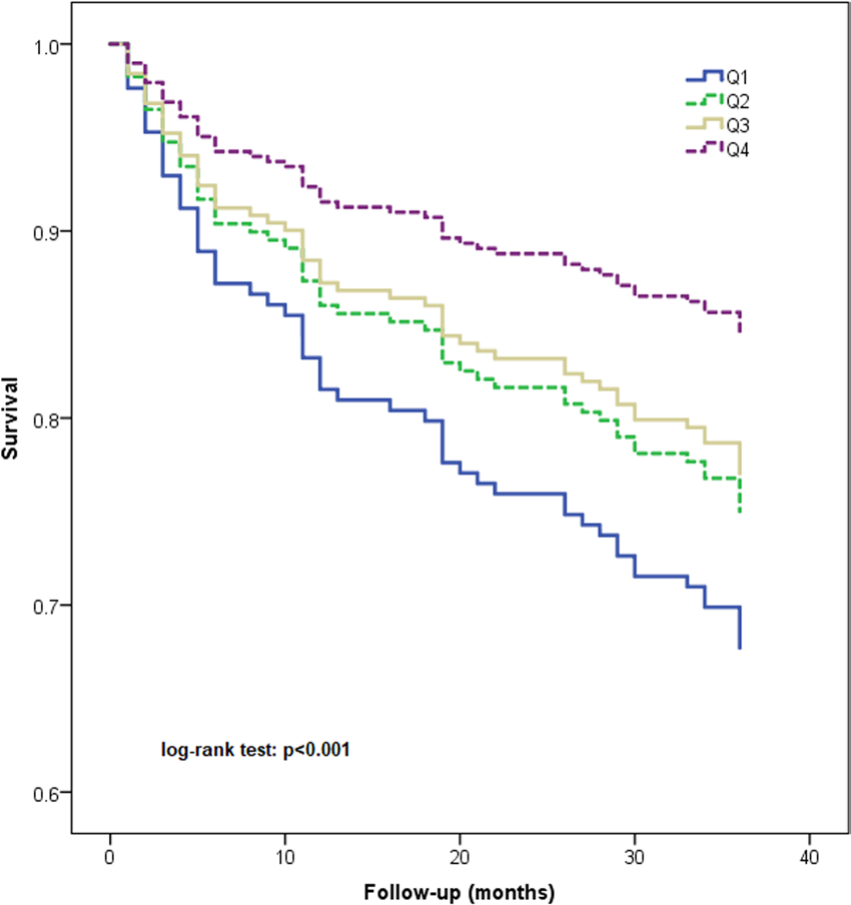
Survival curves of the study population according to the quartiles of sarcopenia index.

Figure 3 shows the survival curves of the participants in different quartile groups according to the SI. The log-rank test indicated that the survival curves of the participants in different quartile groups were significantly different (p < 0.001).

### Association between the SI and readmission

During the 3-year follow-up, a total of 179 participants (75.2%) were readmitted at least one time. The readmission rate according to the quartiles of SI was 84.7%, 78.3%, 71.7% and 66.1% in the Q1, Q2, Q3, and Q4 groups, respectively.

Table 3 shows the association between the SI and readmission according to Cox Regression Models. The SI was not significantly associated with readmission no matter it was treated as either a continuous variable (HR per 1-SD = 0.97, 95% CI: 0.77–1.25) or a categorical data (Q1: HR = 1.23, 95% CI: 0.77–1.99; Q2: HR = 0.97, 95% CI: 0.58–1.61; Q3: HR = 1.10, 95% CI: 0.68–1.76; Q4: reference).

**Table 3:** Association between sarcopenia and readmission (3-year follow-up) according to Cox Regression Models adjusted for potential confounders.

	Unadjusted	Model 1	Model 2	Model 3
Sarcopenia index per 1-SD	0.87 (0.75–1.01)	0.90 (0.78–1.06)	0.96 (0.82–1.12)	0.97 (0.77–1.25)
Quartile of sarcopenia index				
Q1	1.68 (1.11–2.56)	1.40 (0.90–2.18)	1.33 (0.85–2.07)	1.23 (0.77–1.99)
Q2	1.43 (0.93–2.19)	1.29 (0.83–2.01)	1.11 (0.70–1.75)	0.97 (0.58–1.61)
Q3	1.17 (0.76–1.80)	1.03 (0.66–1.61)	1.03 (0.61–1.62)	1.10 (0.68–1.76)
Q4	1 (reference)	1 (reference)	1 (reference)	1 (reference)

Data are presented as hazard ratios (95% confidential intervals). Sarcopenia index was treated as both a categorical variable (using quartile cutoff points) and a continuous variable (per 1-SD), separately.

Q stands for sarcopenia index: Q1 is the lowest quartile and Q4 is the highest quartile. Cutoffs for sarcopenia index are Q1 < 63.9, Q2 63.9-74.3, Q3 74.3-84.2, Q4 > 84.3.

Model 1: adjusted for age and gender. Model 2: adjusted for age, gender, depression and cognitive impairment. Model 3: adjusted for age, gender, depression, cognitive impairment, nutrition status, hemoglobin, body mass index, calf circumference, gait speed, and handgrip strength.

**Figure 4:**
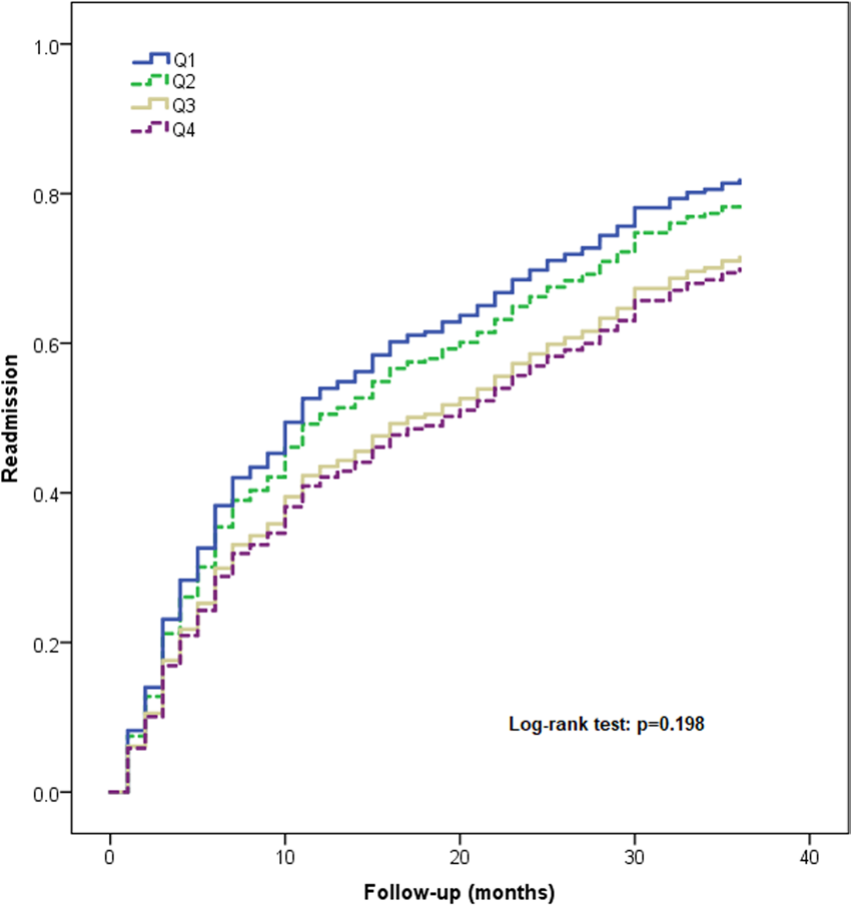
Kaplan-Meier curves for readmission according to the quartiles of sarcopenia index.

Figure 4 shows the Kaplan-Meier curves for readmission of the participants in different quartile groups according to the SI. The log-rank test indicated that this difference between the curves was not statistically significant (p = 0.198).

## Discussion

As we aforementioned, due to the defects of current diagnostic methods for sarcopenia, the validation of a specific biomarker as the surrogate marker of sarcopenia is an attractive method^25^. As a newcomer in this field, the SI and a very similar biomarker (serum creatinine/cystatin C ratio) are of low cost, high accessibility, and repeatability^26^, therefore, they have been studied in different populations since being introduced in 2015^16–19,27^.

Only two previous studies addressed the association between the SI and mortality^16,27^. In a retrospective study conducted in adult ICU patients, the SI was related to in-hospital and 90-day mortality^16^. Recently, another retrospective study also found that a lower SI was associated with a significantly higher risk of mortality among adult ICU patients^27^. Our finding that the SI was associated with long-term mortality among hospitalized older patients was in line with these findings. However, more prospective studies are needed before a robust conclusion can be drawn.

No previous study addressed the association between the SI and readmission. Our study showed that the SI was not associated with readmission in older inpatients for the first time. However, a recent prospective study demonstrated that the SI can be used as a predictor of hospitalization among patients with chronic obstructive pulmonary disease^28^.

Moreover, the serum creatinine/cystatin C ratio has been applied for other purposes in different populations. For example, in a retrospective study, Suzuki *et al*.^29^ reported that serum creatinine/cystatin C ratio appeared to be a predictive marker for chemotherapy-related adverse effects in patients with lung cancer. In another multicenter prospective study, Komorita *et al*.^30^ applied serum creatinine/cystatin C ratio in patients with type 2 diabetes and regularly attending teaching hospitals or diabetes clinics. They found that a lower creatinine/cystatin C ratio was a significant risk factor for fractures.

Putting all these findings together, we may argue that the SI (or serum creatinine/cystatin C ratio) might serve as a potentially valuable biomarker for predicting various adverse outcomes in different populations. However, it is noteworthy that the predictive value of the SI for adverse outcomes does not necessarily mean that it can be applied as a surrogate marker of sarcopenia. In fact, the SI was applied as a malnutrition screening tool in a retrospective study conducted in Mayo Clinic ICU patients^27^. A previous study demonstrated that the SI correlated with CT-derived skeletal muscle mass in adult ICU patients^19^. Similarly, the serum creatinine/cystatin C ratio correlated with DXA-derived skeletal muscle mass in kidney transplant recipients^31^. Furthermore, the serum creatinine/cystatin C ratio was recommended by Osaka *et al*.^32^ as a surrogate screening marker for sarcopenia in patients with type 2 diabetes. However, in a recent prospective study, our team reported that the SI cannot accurately detect either BIA-defined skeletal muscle mass or sarcopenia in urban community-dwelling older people with normal kidney function^18^. The discrepancy of these findings may due to the heterogeneity of the study populations and the methods to measure skeletal muscle mass. It seems that the SI is more sensitive in patients with more serious diseases and with a higher pretest probability of sarcopenia. However, based on the current evidence, whether the SI can be applied to estimate skeletal muscle mass (or sarcopenia) or not (especially among older adults) remains unclear, further studies are therefore warranted.

Interestingly, our study found a positive correlation between the SI and handgrip strength (or gait speed). This finding implies that the SI might be related to muscle function. However, we did not compare the SI with “gold” diagnostic criteria of sarcopenia such as the European Working Group on Sarcopenia in Older People (EWGSOP)^8^ or Asia Working Group for Sarcopenia (AWGS)^9^. Further studies were needed to determine the overall diagnostic accuracy of the SI for detecting sarcopenia, as well as the optimal cut-off points in hospitalized older adults.

Some limitations of our study need to be addressed. First, we did not adjust for some important confounders, such as frailty and activities of daily living. This may induce bias in our results. Second, the sample size of our study is relatively small, especially women. Third, it should be cautious to generalize our results into other ethnic populations or different clinical settings. Fourth, the readmission data were only based on self-reported information, which might be influenced by recall bias. Last, because an eGFR of 90 ml/min/1.73 m2 is the cut-off line of normal kidney function and mildly decreased^33^, we excluded patients with an eGFR < 90 ml/min/1.73 m^2^ (n = 40, 12.8%) from this study to minimize the impact of renal function on the results. Therefore, our results should not be used in older patients with an abnormal kidney function.

## Conclusion

The SI based on serum cystatin C and creatinine is associated with long-term mortality but not readmission in a study population of hospitalized Chinese patients. However, whether the SI really reflects sarcopenia (or something else such as malnutrition) remains controversial. Further prospective studies are therefore needed to compare the SI to sarcopenia per se for predicting important clinical outcomes, such as quality of life and activities of daily living among older adults who living in different settings. Diagnostic accuracy studies of the SI against the classic diagnostic criteria of sarcopenia (or CT- or MRI-derived skeletal muscle mass) among older adults are also needed. There is still a long way to go before the SI can be applied in clinical practice to estimate either skeletal muscle mass or sarcopenia among older adults.

## Data Availability

The datasets generated during and/or analyzed during the current study are available from the corresponding author on reasonable request.
